# SPAN Website for Remote Intervention with Autistic Adolescents and Young Adults: Feasibility and Usability

**DOI:** 10.3390/children10091514

**Published:** 2023-09-06

**Authors:** Liron Lamash, Eynat Gal, Einat Yaar, Gary Bedell

**Affiliations:** 1 Department of Occupational Therapy, University of Haifa, Mount Carmel, Haifa 3498838, Israel; lironlamash@gmail.com (L.L.); einatyaar@gmail.com (E.Y.); 2 Department of Occupational Therapy, Tufts University, Medford, MA 02155, USA; gary.bedell@tufts.edu

**Keywords:** youth, autism spectrum disorder, telehealth, social participation

## Abstract

Adolescents and young adults (AYA) with autism spectrum disorders (ASD) report less functional independence and social participation than their neurotypical peers. Remotely delivered interventions may allow autistic AYA to promote their independence, social participation, and wellbeing as they transition to adulthood. Social Participation and Navigation (SPAN) is a technology-based remotely delivered intervention initially developed for AYA with acquired brain injuries. The SPAN (website, application, and intervention manual) was modified to address the needs of AYA with autism (SPAN-ASD). This study examined the SPAN-ASD website and web application’s feasibility and usability. Participants comprised 12 autistic AYA and 18 practitioners (all occupational therapists) with more than 1 year of experience in working with autistic AYA. All navigated the SPAN-ASD website and goal-management application. Practitioners completed the SPAN-ASD components and the Usefulness, Satisfaction, and Ease of Use questionnaires; AYA completed the System Usability Scale. The practitioners’ average feasibility scores ranged from 4.30 to 4.68 (high); the overall usability score was 5.77 (good). The autistic AYA rated SPAN-ASD as a good, acceptable, and useful tool. Content analysis and item-level ratings indicated some needed improvements. Practitioners and autistic AYA perceived the SPAN-ASD website and application as highly feasible and usable, with excellent potential for technology-supported interventions.

## 1. Introduction

Adolescence is an important developmental phase marking the transition from childhood to adulthood [1]. During this period, adolescents and young adults (AYA) begin to take more control of their actions and personal decision making, striving for autonomy and independence [2,3,4]. The adolescence and young adulthood years especially challenge individuals with autism spectrum disorder (ASD).

Characteristics of autism spectrum disorder, a neurodevelopmental disorder spanning a lifetime, include social communication deficits, deficits in interactions across contexts, and restricted repetitive behavior patterns [5]. Autistic AYA often report below-age functional independence skills and the need for caregiver support or supervision to perform routine tasks and activities [6,7,8,9]. Evidence suggests that their participation in activities involving social interactions (i.e., social participation) is lower compared to AYA with neurotypical development [10,11] or other neurodevelopmental disabilities [12]. In addition, autistic AYA often need or request problem-solving support to participate in community and social activities, education, work, and housing [13,14]. These challenges, combined with insufficient environmental and societal support, contribute to their struggles transitioning into adulthood and highlight the need for interventions that promote their independence, social participation, and wellbeing [15,16,17,18]. Moreover, autistic individuals have expressed that a key priority is research and interventions aiming to develop living skills and improve daily life participation (e.g., [19,20]).

An accumulation of promising evidence has highlighted the important potential benefits of using accessible technology devices (e.g., mobile phones, tablets, and computers) to deliver effective interventions for autistic individuals [21,22,23,24,25]. The use of remote interventions has significantly increased recently, particularly during the COVID-19 pandemic [26,27], but the effectiveness and efficiency of service-delivery systems are still being examined [28]. Moreover, little evidence has been reported on the effects of remotely delivered interventions for autistic AYA [29,30], specifically to promote their independence and participation goals [31,32].

### 1.1. Social Participation and Navigation

Social Participation and Navigation (SPAN; www.spanprogram.com) is a promising technology-based remotely delivered intervention that might benefit autistic AYA. It was first designed in the USA to improve the social participation of AYA with acquired brain injury (including brain-tumor and traumatic brain injury survivors) via their creation and achievement of personal goals. Initially, the SPAN comprised an iPhone mobile application with brief “Tips and Topics” (e.g., setting goals and planning to achieve them, social participation, self-regulation, and staying positive) and video-conferencing with a college-student coach who was trained and supervised [33]. 

The initial SPAN iPhone mobile application experienced technical problems, leading to the development of a new website with “Tips and Topics” and a web-based goal-planning application [33,34,35,36]. The SPAN intervention process included up to 10 weekly coaching sessions aimed at identifying and achieving feasible social-participation goals. With the goal-planning application, users identified strategies and created step-by-step plans to achieve each goal. Coaches provided ongoing support for the participants to identify hindrances, supports, and resources they could use to achieve their goals [33,34,35,36,37].

Preliminary results from implementing the SPAN program for adolescents with acquired brain injury indicated high satisfaction with the technology. Participants successfully met virtually with their coaches and used the technology to develop and achieve social participation goals [33,34,35,36,37]. Although further research is needed to address SPAN’s efficacy with other users and coaches, the intervention has excellent potential as an innovative intervention, supported by technology, for autistic AYA.

### 1.2. Adapting SPAN for Autistic AYA

In a prior study, we conducted a formative evaluation to inform the modifications of the initial SPAN intervention manual, website, and web application that autistic AYA would find useful [38]. In that study, 15 researcher and clinician stakeholders from the USA and Israel who worked with autistic AYA provided feedback and recommendations about what content and features to preserve, add, and/or modify from the original SPAN, who should deliver it, and who would benefit most from the modified SPAN (SPAN-ASD). For example, it was recommended that the SPAN-ASD should continue to be a customized intervention promoting the personal goals of autistic AYA that could be supported remotely and in a short period (up to 10 sessions). However, it was suggested that various personal (e.g., academic, self-care, and daily living activity) goals could be addressed in addition to social participation goals. The results also suggested that the SPAN-ASD program would most benefit autistic AYA 12 yrs. and older (verbal and nonverbal), who could read and write (allowing communicate with practitioners) and use all website and application options (e.g., navigating, writing goals, tasks, and strategies). Finally, it was recommended that practitioners (e.g., occupational therapists [OTs], speech therapists, emotional therapists, and educational staff) experienced in working with autistic AYA should deliver the intervention rather than the trained supervised college-student coaches who delivered the original SPAN intervention for AYA with acquired brain injury. 

Following the formative evaluation, the SPAN-ASD intervention procedural manual, website, and application were created in English and Hebrew. Similar to the original program, the SPAN-ASD intervention includes three main components: 

Weekly videoconferencing meetings (via Zoom or Google Meet) with a clinician who helps the participant identify and set goals, identify strengths and supports, generate strategies, develop their action plan, and refine it as needed. 

A website with accessible informative web pages on intervention-related topics, such as being an autistic AYA, strength-based interventions, the importance of independence, social participation, and metacognitive strategies. The personalized area (web application) is password protected. It includes options for setting goals, planning action steps, adding times and notifications, and entering existing and new strategies into a strategies bank. 

The intervention manual, a structured and detailed guide for practitioners to use during the intervention, includes relevant information (e.g., theoretical basis and website-user guidelines). The manual outlines the customized intervention, including coaching principles from cognitive and strength-based approaches (e.g., general session structure, recommended dialogue topics for meetings, and documentation pages). 

### 1.3. The SPAN-ASD Website and Web Application

**Figure 1:**
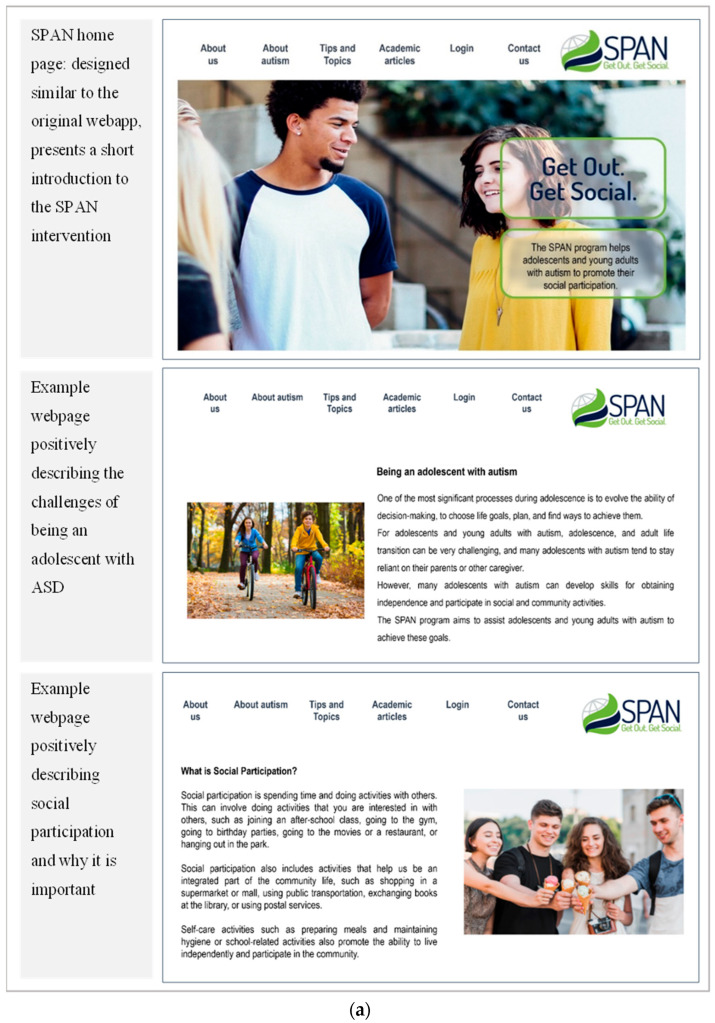

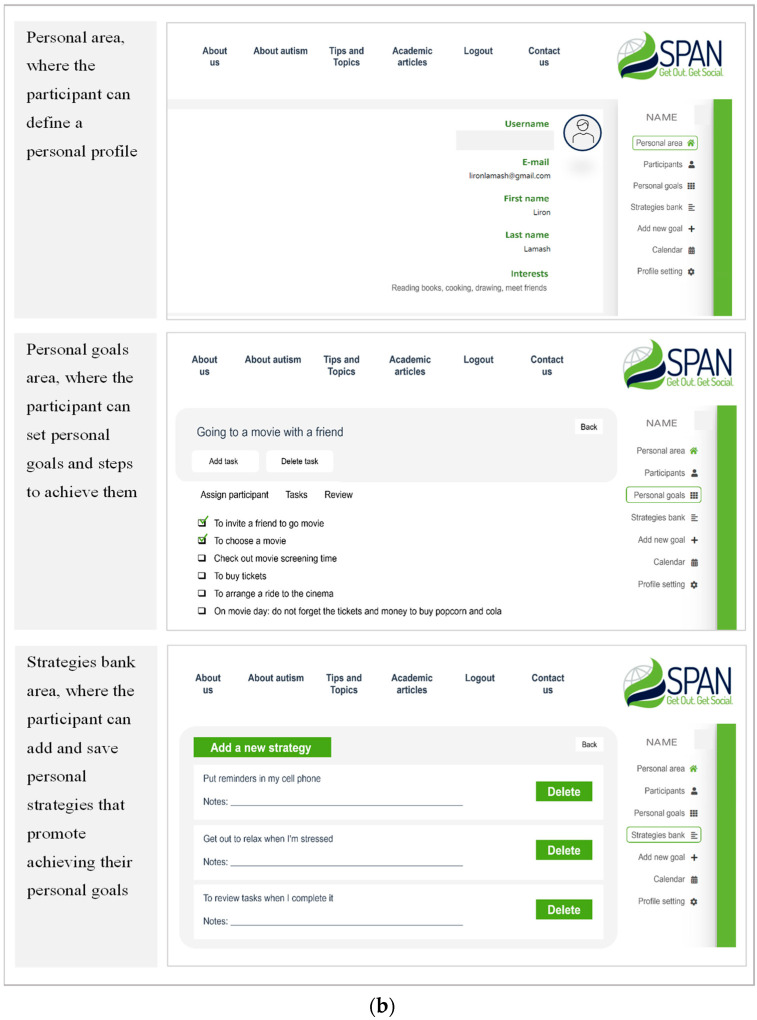
(**a**). Example Social Participation and Navigation (SPAN-ASD) open-area screenshots. (**b**). Example Social Participation and Navigation (SPAN-ASD) personal-area screenshots.

The design and features of the SPAN website and application (login-personalized area) designed for AYA with acquired brain injury informed the development of the SPAN-ASD website and application. The current SPAN-ASD website (https://en.spanprogram.co.il) was developed using WordPress, available in English and Hebrew. Its general design includes web pages with free access to content (e.g., program information, population-relevant content, tips, and topics; see Figure 1a for examples). The SPAN-ASD website also includes a web application for the personalized area that requires a username and password. In the SPAN-ASD web app, the users (practitioners and autistic AYA) manage the goal-planning process, review the plan, and create a strategies bank (see Figure 1b for examples). 

### 1.4. Research Aims

This current study aimed to examine the feasibility (of the content, operations, and structured/sequenced experience) and usability (including ease of learning and use, satisfaction, and usefulness) of the newly designed SPAN-ASD website and application in Israel (in its Hebrew version) with autistic AYA and practitioners who work with autistic AYA. Subsequent studies will include a pilot and implementation study using the SPAN-ASD’s full version in Israel (i.e., all key components).

## 2. Materials and Methods

### 2.1. Participants

#### 2.1.1. The Practitioners

The inclusion criterion was least 1 year of experience in working with autistic AYA. No exclusion criteria were defined. All 18 health care practitioners who agreed to participate happened to be Israeli OTs. These participants completed a demographic questionnaire developed for this study to describe the sample. The sample included 18 female practitioners aged 24 to 48 yrs. (*M* = 34.78 yrs., *SD* = 7.26). Most (*n* = 13, 72%) worked mainly with autistic AYA at Level 1 (i.e., requiring some support) per the *Diagnostic and Statistical Manual of Mental Disorders*-5th edition (*DSM-5*) [5] integrated into mainstream schools. Participants’ clinical experience ranged between 1 and 23 yrs. (*M* = 10.00 yrs., *SD* = 7.09), with 1 to 10 yrs. (*M* = 4.00 yrs., *SD* = 3.25) experience working with autistic AYA. Most had postgraduate degrees (BA/BOT; 55.6%, *n* = 10); five (27.8%) had Master’s (MA/MSc) degrees, and three (16.7%) had PhD degrees. They reported their frequency of technology use in intervention sessions on a scale from 0 (not at all) to 5 (extremely often). Although their pre-COVID-19 use was extremely low (*M* = 1.28, *SD* = 1.57), they reported significantly more technology use during the pandemic (*M* = 2.28, *SD* = 1.97), *t*(17) = −2.92, *p* < 0.01, *d* = 1.46. 

#### 2.1.2. The Autistic AYA

The 12 autistic Israeli AYAs, recruited through their educational settings in a convenience sample, were aged 12 to 21 yrs. (*M* = 16.2, *SD* = 3.3). Nine identified as males (75%), and three identified as females. Six (50%) were students in middle school, and six (50%) were students in high school. Nine (75%) participants studied in a special education school for autistic students up to age 21, and three (25%) studied in a special education class for autistic students integrated into a mainstream school. 

As verified by their parents’ report, all AYA participants had a formal ASD (Level 1) diagnosis based on the *DSM-5* criteria [5] and received an ASD diagnosis when they were between the ages of 3 and 10 (*M* = 4.6, *SD* =2.8). All met the conditions for participation in the SPAN intervention (e.g., having writing and reading abilities, verbal communication ability, skills and experience in web navigation and using applications, and access to technology/internet). No exclusion criteria were defined. 

### 2.2. Data Collection

Practitioners completed two questionnaires following their guided use and review of the SPAN-ASD website and application: the SPAN-ASD Components Questionnaire and the Usefulness, Satisfaction, and Ease of Use Questionnaire (USE [39]). Autistic AYA completed the System Usability Scale (SUS [40]).

#### 2.2.1. The SPAN-ASD Components Questionnaire

The semistructured questionnaire developed for this study included 23 questions related to the SPAN-ASD website and application: Ease of navigation included four items rated on a five-point scale from 1 (not easy at all) to 5 (very easy); relevance of content included five items rated from 1 (not relevant at all) to 5 (very relevant); ease of performing operations included six items, such as “How easy is it to set a schedule for a task?” rated from 1 (not easy at all) to 5 (very easy); visual format included three items, such as “To what extent are the images on the SPAN-ASD website suitable for autistic AYA?” rated from 1 (not at all) to 5 (very much); and necessity of components included five items rated from 1 (not needed at all) to 5 (very much needed). The questionnaire also asked several open-ended questions to obtain feedback about the webpage content and other populations that might benefit from SPAN-ASD and to provide recommendations.

#### 2.2.2. Usefulness, Satisfaction, and Ease of Use Questionnaire 

Participants completed the USE questionnaire [39], a 30-item measure that assesses a product’s or service’s perceived usability. For each item statement, the degree of agreement is ranked on a seven-point scale from 1 (strongly disagree) to 7 (strongly agree). The USE includes a total usability score derived from the average of all items and four group-usability scores from the average of items in each dimension: ease of use (11 items) and learning (four items), satisfaction (seven items), and usefulness (eight items). The USE has good face validity and a Cronbach’s alpha of 0.98, indicating the high internal consistency reliability of its overall score [39,40].

#### 2.2.3. System Usability Scale

The SUS [41] is a widely used self-report questionnaire allowing an expansive view of subjective usability assessments [42]. It consists of 10 phrases regarding the evaluated technology: even phrases have negative tones, and odd phrases have positive tones. The items are rated on a five-point Likert scale from 1 (strongly disagree) to 5 (strongly agree). The participants’ scores for each question are converted to a scale of 0 to 4, respectively, and summed. Then, they are multiplied by 2.5, converting the raw scores into a range from 0 to 100 (higher scores signal better usability). Extensive research indicated that SUS scores above 68 could be considered above-average usability [43,44]. The SUS has evidence of high internal consistency reliability (α = 0.91 [43]). 

### 2.3. Procedure

The University of Haifa Ethics Committee (approval No. 182/19) and the Israeli Chief of Science Office in the Ministry of Education (approval No. 11734) approved this research.

We recruited the health care practitioners using convenience sampling through advertisements targeting social-media interest groups of practitioners serving autistic AYA (all the participants who expressed a desire to participate happened to be OTs). We emailed the practitioners who expressed a desire to participate a link to the SPAN-ASD website, login information for the web application (username and password for the personalized area), and a detailed instruction guide. The instruction guide included tasks that allowed users to practice and experiment with the website and application components (e.g., navigating the information pages, logging into the personalized area, adding a client, and setting intervention goals). During the experiential phase, the practitioners completed the online questionnaires using Google Forms. 

A researcher met the autistic AYA in their school and provided them the link to the SPAN-ASD website and login information for the personalized area. After providing a short explanation and demonstration given, the researcher asked the AYA to review the website and experience the SPAN ASD application features independently (e.g., create a profile, set a goal, set steps, and add strategies to the strategies bank). They used the SPAN-ASD website and application weekly for 3 weeks (supervised by the researcher) and then rated their usability independently via the SUS [40].

### 2.4. Data Analysis

We conducted the data analysis using IBM SPSS (ver. 27.0). Descriptive statistics (frequencies, ranges, means [*M*], and standard deviations [*SD*]) were used to examine the demographics and the feasibility and usability scores. Content analysis was conducted that mainly summarized feedback and suggestions for improvement. 

## 3. Results

Most usability findings came from the practitioners; thus, their results will be presented first, followed by those from the autistic AYA. This section first presents the ranges, means, and standard deviations for the feasibility and then the item-level frequencies for each of the five categories from the SPAN-ASD Components Questionnaire. The practitioners’ recommendations for additional adaptations for autistic AYA are also presented for selected categories. Then the ranges, means, and standard deviations for the SPAN-ASD website and application usability from the USE questionnaire (for overall usability score and four usability dimensions from practitioners) and the SUS questionnaire (from autistic AYA) are presented.

### 3.1. Website and Web Application Feasibility (SPAN-ASD Components Questionnaire): Practitioners’ Responses

#### 3.1.1. Category Total Feasibility

**Figure 2:**
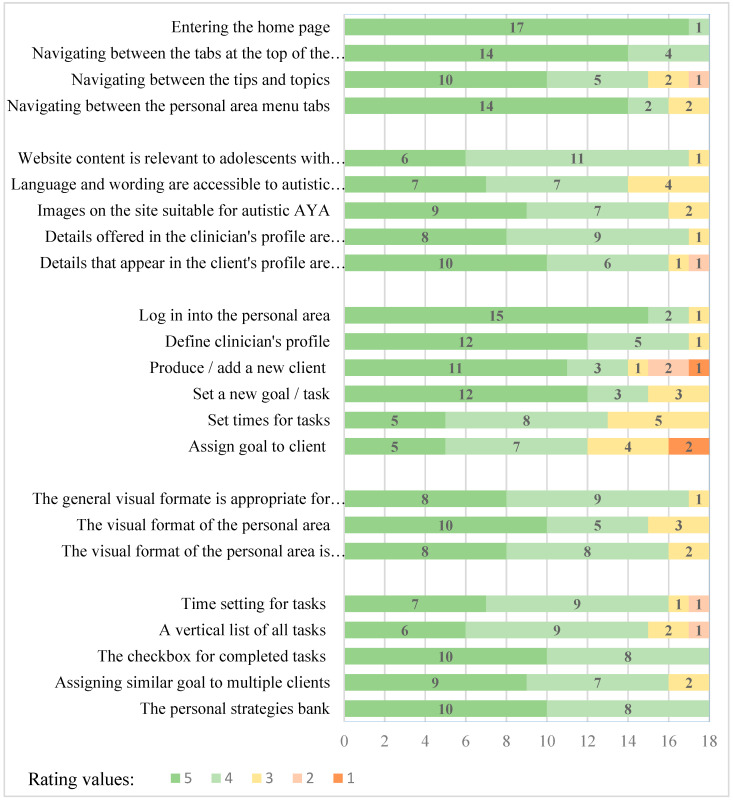
Rating frequency in the SPAN-ASD Components Questionnaire (*N* = 18).

The total feasibility mean score of the SPAN-ASD website and application was 4.34 (*SD* = 0.30); item mean scores ranged between 4.30 and 4.68 (above high feasibility; Table 1). Figure 2 presents the frequency of each rating level in each of the five categories. 

#### 3.1.2. Item-Level Feasibility 

Items in all categories were also rated on a scale of 1 (not feasible) to 5 (very feasible).

**Ease of navigating the website and web application**. This category included four items on how easily the SPAN-ASD website and application were navigated (e.g., entering the home page or navigating between web pages/tabs). The mean item scores in this category ranged between 4.33 and 4.94 (feasible and very feasible). Most items were rated as very easy to navigate (i.e., rated 5 or 4). However, the navigation between the tips and topics was rated slightly less accessible. 

**Content relevance.** This category included five items on the relevance of information on the SPAN-ASD website, issues pertaining to the Tips and Topics section, details in the user’s profile, overall language, wording, and images. The mean item scores ranged from 4.17 to 4.39. Most items were rated as very relevant (i.e., rated 5 or 4). However, four participants rated the wording and language as moderately appropriate to autistic AYA. In addition to those rated items, the practitioners were asked to mark content that seemed relevant to the intervention (from a list of the existing content). The content they most frequently rated as relevant addressed developing strategies to achieve personal goals (*n* = 17), developing autonomy (*n* = 17), and time organization (*n* = 17), followed by social participation and functional independence (*n* = 16), being an autistic AYA (*n* = 15), and using a strength-based approach (*n* = 14). Although eight practitioners suggested that the existing content was sufficient and the website should not be overloaded with additional content, seven suggested adding content such as references to romantic and sexual relationships, self-advocacy, and autistic people’s rights. Additional suggestions included adding links to relevant social community activities, relevant social groups on social media, and organizations and associations relevant to autistic people.

**Ease of performing operations**. This category included six items about the ease of performing actions within the SPAN-ASD web application (the personalized area). The main actions rated included logging in, defining a user profile, adding a new client, and setting goals and tasks. The mean item scores ranged from 3.72 to 4.78. In this category, the response range was wider, indicating that adding a new client, assigning a goal to a client, and setting a time for the task were more complex for some practitioners.

**Visual format.** This category included three items about the SPAN-ASD website and application’s visual format and appearance (e.g., color scheme, visual load, font type, and size). The mean item scores ranged between 4.33 and 4.39. Most practitioners rated them as having appropriate visibility for autistic AYA (i.e., 4 or 5). Additional visual adaptations for autistic AYA suggested by practitioners included increasing the font of the web application menu (on the screen’s left side) and increasing the visual contrast of a selected tab from the other options in the menu.

**Components’ necessity.** This category included five items about the necessity of features available in the web application (the personalized area) to help the users manage their personal goals. These features included setting times and reminders, monitoring tasks, and creating a strategy bank. The mean item scores ranged between 4.11 and 4.56 (above needed). In this category, all web application components were rated as very needed. An additional application component suggested was to add an option for practitioners to assign goals for multiple clients (e.g., for group intervention).

### 3.2. Website and Web Application Usability (USE Questionnaire): Practitioners’ Responses

We assessed the website and application usability via the USE questionnaire [39] completed by the practitioners. The overall score for the SPAN-ASD was 5.77 (of 7.00), indicating good usability. The SPAN ease of use was rated relatively the lowest (*M* = 5.46, *SD* = 0.81), and ease of learning was ranked the highest (*M* = 6.03, *SD* = 0.75). Table 2 presents the ranges, means, and standard deviations of the four usability dimensions.

### 3.3. Website and Web Application Usability (SUS Questionnaire): Autistic AYA Responses

The SUS total scores ranged from 50.0 to 92.5 (*M* = 75.83, *SD* = 12.72), indicating that the SPAN-ASD is a good, acceptable, and useful tool [43]. Furthermore, of the 12 participants, nine (75%) rated the SPAN above average. All the autistic AYA rated the item about their confidence using the SPAN (Item 9) as the highest; however, they indicated some need to learn more about the website before they could use the SPAN (Item 10). Table 3 shows the SUS raw mean scores by item: For odd items, higher scores indicate greater usability (high agreement with the statement); for even items, lower scores indicate greater usability (less agreement with the statement). 

## 4. Discussion

This study examined the feasibility and usability of the newly developed SPAN-ASD website and application (Hebrew version) with 12 autistic AYA and 18 practitioners who work with autistic AYA. It is important to note that a parallel process of examining the feasibility and usability of the SPAN technology is also being conducted in the English version in the USA. 

Despite varying seniority, academic backgrounds, and technological experience, the practitioners’ feedback indicated that they perceived the SPAN-ASD website and application as highly feasible and usable. The practitioners also suggested improving some technological components and adding some content to the website and application that informed subsequent updates. According to the autistic AYAs’ perspectives, the usability findings also indicate the high usability of the SPAN website and app. The findings from the practitioners’ perspectives will be discussed first, followed by findings from the autistic AYAs’ perspectives.

### 4.1. Ease of Navigating and Performing Operations 

Principles culled from the literature suggest that websites for the ASD population should include simple, consistent, and intuitive navigation and present only features relevant to the users’ needs [45,46]. The SPAN-ASD’s personalized goal-management area was designed with great simplicity to reduce distractions and increase autistic users’ engagement. It uses a neutral color palette, blank backgrounds, static images, and no interfering images or items. 

The practitioners rated the SPAN-ASD website and application (the personalized goal-management area) as relatively easy to navigate, confirming its accessibility for autistic AYA. However, the “Tips and Topics” section was designed as a list of links, a style different from the other open-area web pages. Three of the 18 practitioners rated this section as more difficult to navigate than the personalized area. Future improvements to the website will focus on “Tips and Topics” navigation and some personalized area operations that received lower ratings (e.g., adding new clients or assigning goals).

### 4.2. Content Relevance and Components’ Necessity 

The SPAN-ASD website includes an open-access webpage for all users. The primary tabs briefly describe the SPAN program, ASD, being an autistic AYA, and “Tips and Topics.” The “Tips and Topics” tab introduces a list of links to various websites on issues relevant to the intervention program. There was concordance between the practitioners’ ratings of this section and evidence in the literature about interventions for autistic AYA.

In general, the practitioners highly rated the relevance of the website content. Almost all rated the “importance of developing autonomy” as the most needed and essential content. Recent literature has pointed to the importance of developing the autonomy of autistic AYA as a basis for transitioning to work and community life [47,48,49]. The practitioners also rated “social participation” and “functional independence” as very relevant content, which is consistent with reports that these variables should be the main targeted goals for autistic AYA [50,51,52]. The tips for metacognitive strategies to achieve goals and time organization were also rated as necessary and vital content, consistent with reports that these areas are core abilities of daily functioning that should be highlighted during interventions with autistic individuals [53,54,55,56,57].

Most practitioners noted the importance of presenting content pages on how to handle an ASD diagnosis and using an approach based on the user’s strengths. These findings are consistent with stakeholders’ recommendations from the formative evaluation study [38] and reports in the literature that emphasized using strength-based interventions focused on harnessing or leveraging strengths despite challenges (versus remediating deficits or impairments) to improve outcomes for autistic individuals, especially when delivered via technologies [52,58,59].

### 4.3. Visual Format

The SPAN-ASD website and application were developed based on key stakeholders’ recommendations from the prior formative evaluation study [38]. Prior recommendations resulted in designing the SPAN-ASD website and application with short, focused, and simple text and a consistent pleasant visual design. These recommendations were congruent with the literature addressing technology design for autistic individuals. That research recommended using color contrast between background and objects to distinguish items, simple language with succinct paragraphs [45,60,61], and realistic, compatible, easily recognizable images [62,63].

Practitioners in this study rated the SPAN-ASD website’s and app’s general visual format as highly feasible. Their overall high ratings reflect the integration of adapted design elements into the revised website and application. Those elements are consistent with the Academic Autism Spectrum Partnership in Research and Education’s recommendations for simple physical and cognitive accessibility for autistic web users [46]. 

Although we received the most feedback from practitioners, we also received feedback from autistic AYA exposed to the SPAN website and application via the shorter SUS questionnaire [43,64]. The autistic AYA rated the SPAN-ASD usability as above average. They all “felt very confident using the product” (Item 9, all rating 4) and rated the SPAN-ASD website and application as a consistent product (i.e., rating the item, “I thought that there was too much inconsistency in this product” less than 1 = disagreement). These findings correspond with the practitioners’ feedback and support the design of the SPAN-ASD website and application and its application for autistic AYA.

In sum, both practitioners and autistic AYA rated the SPAN-ASD website and application as highly feasible and useful for autistic AYA. The feedback confirmed key stakeholder recommendations from the prior formative assessment phase [38] and informed what will be improved in the SPAN-ASD website and application modifications. The positive results were probably due to using an iterative development process similar to that used for the original SPAN website and application designed for AYA with acquired brain injury, a population who also have challenges with social and executive functioning and self-management [33,36,37,65]. 

Although the findings provide preliminary evidence supporting the feasibility and usability of the new SPAN-ASD website and application, the study limitations should be considered. Although the SPAN technology was designed for use by practitioners from various disciplines (e.g., speech therapists, emotional therapists, and teachers), only OTs volunteered for and were included in this study. Further research should examine the feasibility and usability of the SPAN technology among other practitioners who provide continuous service to autistic AYAs. In addition, the current study examined the Hebrew version of the SPAN technology. Additional investigation (in process) will assess the more international version in English. Although the practitioners provided valuable information from their perspectives, the information about the autistic AYAs’ perspectives, albeit very positive, was based solely on the brief SUS [40]. Future studies should include more comprehensive, detailed, and targeted feedback from autistic AYA. Moreover, this study focused only on the website and application. Thus, a pilot implementation trial will be conducted to examine the efficacy of the full SPAN-ASD components (website, application, and intervention program) further and its potential benefits related to promoting participants’ personal functional independence and social participation goals.

## Figures and Tables

**Table 1:** SPAN-ASD Components questionnaire: practitioners’ responses.

SPAN-ASD Website Component Category	Range	*M*	*SD*
a. Ease of navigating the website	3.75–5.00	4.68	0.38
b. Relevance of content	3.40–4.80	4.32	0.41
c. Ease of performing operations	3.50–4.83	4.30	0.42
d. Visual format	3.33–5.00	4.37	0.58
e. Necessity of components	3.60–5.00	4.37	0.41
Total score	3.86–4.76	4.34	0.30

Note. All items were rated on a five-point scale from 1 (not feasible) to 5 (very feasible).

**Table 2:** Usefulness, Satisfaction, and Ease of Use (USE) questionnaire: Practitioners’ responses.

USE Dimension	Range	*M*	*SD*
Usefulness	4.50–6.75	5.89	0.67
Ease of use	3.73–6.82	5.46	0.81
Ease of learning	4.50–7.00	6.03	0.75
Satisfaction	4.71–7.00	5.97	0.68
Total score	4.73–6.63	5.77	0.56

**Table 3:** System Usability Scale (SUS): autistic AYA responses.

	Item	Range	*M*	*SD*
1.	I think that I would like to use this product frequently	1–4	3.00	1.28
2.	I found the product unnecessarily complex	0–4	1.17	1.40
3.	I thought the product was easy to use	1–4	3.17	1.11
4.	I think that I would need the support of a technical person to be able to use this product	0–4	1.33	1.50
5.	I found that the various functions in this product were well integrated	2–4	3.17	1.03
6.	I thought that there was too much inconsistency in this product	0–4	0.92	1.24
7.	I would imagine that most people would learn to use this product very quickly	1–4	3.33	0.98
8.	I found the product very awkward to use	0–4	1.08	1.31
9.	I felt very confident using the product	4–4	4.00	0.00
10.	I needed to learn a lot of things before I could get going with this product	0–4	1.83	1.80

Note. The scores are on a 0- to-four-point scale. For odd items, higher scores indicate greater agreement; for even items, higher scores indicate less agreement. Items are replicated from the SUS, which is freely available; the table was adapted from Brooke [41], ©Digital Equipment Corporation, 1986.

## Data Availability

The data presented in this study are available on request from the author. The data are not publicly available due to ethical restrictions.
